# Towards an Asian registry of wrongful convictions

**DOI:** 10.1016/j.fsisyn.2026.100726

**Published:** 2026-09-02

**Authors:** Yiwen Zhang, Henry Otgaar, Yikang Zhang, Nathanael E.J. Sumampouw, Tsania Isa, Jianqing Wang, Yoshiyuki Nishi, Yun-Ching Ko

**Affiliations:** a Faculty of Psychology and Neuroscience, Maastricht University, Maastricht, the Netherlands; b Leuven Institute of Criminology, Faculty of Law and Criminology, KU Leuven, Belgium; c Max Planck Institute for the Study of Crime, Security and Law, Germany; d Faculty of Psychology, University of Indonesia, Indonesia; e Department of Psychology, Fudan University, China; f Innocence Project Japan, Japan; g Stanford Law School, Stanford University, United States; h Taiwan Innocence Project, Taiwan

**Keywords:** Wrongful conviction, Asia, Documentation platform

## Abstract

Wrongful convictions represent a persistent yet unevenly documented problem across Asian criminal justice systems. While international research has identified common factors contributing to known wrongful convictions, systematic information about wrongful conviction cases in Asia remains fragmented and difficult to compare across jurisdictions. This fragmentation limits cross-jurisdictional learning and comparison of documented cases. This paper synthesizes existing research on contributing factors in documented wrongful conviction cases in Asia and reviews current initiatives addressing such cases. It then showcases an operational map for the Asian Registry of Wrongful Convictions (ARWC), an academic documentation platform designed to record final criminal convictions that were later officially corrected or acknowledged as erroneous. By setting out the registry's scope and finality requirement, its inclusion criteria and how they were developed, its partial harmonization strategy, documentation workflow, and feasibility, the paper offers a concrete next step toward improving transparency and comparative understanding of wrongful convictions in Asia.

A wrongful conviction refers to a conviction imposed on an individual who should not have been found guilty under the law. Most scholarly definitions emphasize that wrongful convictions occur when a person is convicted of a crime they did not commit [1,2]. This includes “no-crime” cases, in which the individual was convicted of an act that never occurred, such as accidents or suicides misperceived as homicide [3]. It also includes cases in which another person committed the offense [4,5].

Wrongful convictions may also arise when the defendant did commit the act but is not legally culpable because a valid affirmative defense, such as self-defense, necessity, insanity, or duress, was incorrectly rejected by the court [6,7]. Legal scholars have further noted that wrongful convictions are often identified when the criminal justice system later acquits, exonerates, quashes, or vacates the conviction, thereby formally acknowledging error^1^ (see Ref. [2]). However, most wrongfully convicted individuals do not receive an exoneration, even when error is present, because legal reversal may require new evidence or may take years [8,9]. Across definitions, wrongful conviction is understood as an injustice and a system failure, reflecting errors in evidence, procedure, or decision-making within the criminal legal system [2].

Wrongful convictions represent a species of miscarriage of justice, where individuals are punished for crimes that they did not commit. In this paper, “wrongful convictions” refers to known or officially documented cases rather than the full, unobserved universe of wrongful convictions. In the United States, the National Registry of Exonerations has recorded more than 3843 overturned convictions since 1989, spanning crimes from minor offences to serious felonies [10]. Similar documentation efforts have emerged elsewhere. In Canada, a national registry of wrongful convictions compiles cases recognized by courts or state authorities. At the regional level, the European Registry of Exonerations (EUREX) collects and standardizes information on exonerations across multiple European jurisdictions, enabling cross-national comparison. These initiatives share a common premise: that systematic, centralized documentation is what turns individual corrected cases into useable knowledge about how convictions fail.

Much of the global attention on wrongful convictions has predominantly focused on issues faced in North America and Europe [[11], [12], [13], [14], [15]]. This skewed Western global attention stresses the need to more systematically examine the topic of wrongful conviction in other parts of the world such as in Asia. Available indicators from Asian jurisdictions suggest the problem is real but officially corrected cases are rare. In China, official statistics indicate an exceptionally low rate of acquittal at trial: 294 defendants were declared not guilty in 2025 ([16]; see also [17]). Regarding the correction of convictions that have already become final, the Supreme People's Court [18] reported 83 cases involving 101 people in which wrongful convictions were corrected through retrial acquittal in 2024. Japan's official post-conviction correction figures remain low in most years. National statistics on decisions to commence retrial show that, aside from an exceptional spike in 2013 ([19]; over 400 retrial-commencement decisions in Road Traffic Act cases linked to improperly operated automated speed-enforcement devices), retrial-commencement decisions have generally been in the single digits annually [20]. Such figures depend heavily on definitions (e.g., whether mass traffic-case retrials are included), and scholars note that publicly visible corrections of wrongful convictions remain limited relative to conviction outcomes [21]. Taiwan has documented 308 retrial-based exonerations since 1991, including seven former death row prisoners, one of whom was exonerated posthumously [22]. In Indonesia, civil society reporting has highlighted multiple cases involving wrongful conviction concerns and procedural violations, though systematic official documentation remains limited [23]. In Singapore, scholarship has discussed cases in which convictions were later corrected, including instances linked to coerced confessions or contested forensic evidence [24,25]. Although official “not-guilty” or exoneration rates remain extremely low [26,27], the true number of innocent individuals caught in the system remains unknown.

Asian countries are equally confronted with significant challenges to accurately address wrongful convictions. Scholars are increasingly noting that wrongful convictions not only inflict deep harm on a social, psychological, and economic level upon exonerees, they also erode public trust in the criminal justice system [12,28]. However, research on *Asian* wrongful convictions is limited compared to that in Western context [14,15]. Unrecorded wrongful convictions across the region likely exceed what official metrics or media coverage can capture [29].

Despite mounting evidence that wrongful convictions occur in numerous Asian jurisdictions, researchers face substantial barriers in obtaining reliable, comparative data [15]. The wider landscape of wrongful convictions, particularly in countries with fewer English-language sources, remains largely unmapped [14]. When possible wrongful convictions in Asia are documented, academic and legal discussions have often exclusively focused on high-profile cases, especially from China [29], Indonesia [30], and Taiwan [31]. While this focus has yielded important insights, such as the impact of coerced confessions on wrongful convictions, it risks overshadowing pressing issues in other Asian jurisdictions like Malaysia or the Philippines, where wrongful convictions are no less concerning but far more difficult to document [15,25]. Few jurisdictions within Asia maintain any centralized official record of exonerations or wrongful convictions. Relevant information is often scattered across court documents, media reports, and disparate government agencies. Even in countries where judicial transparency is improving (e.g., Japan's partial adoption of video-recorded interrogations), consistent, accessible data on exonerations remain scarce [21,32].

While English serves as a dominant language in international legal research, a significant volume of wrongful conviction studies, court rulings, and forensic analyses may be published exclusively in national or regional languages (e.g., Japanese, Korean, Malay, Tagalog). This issue can lead to a filtering effect: only research or case documentation translated into English reaches a global readership, leaving many documented cases unseen outside their own jurisdiction. Collaborative efforts between English-speaking scholars and regional experts are not yet systematic. As a result, even when a case is well documented in local-language sources, it remains effectively unavailable to comparative research: it cannot be located, counted, or compared across jurisdictions. The consequence is that the scale of wrongful convictions in the region is underestimated, and recurring patterns of forensic and evidentiary error remain confined to the jurisdictions in which they occurred. Addressing this gap requires more than the translation of individual studies; it requires shared documentation infrastructure.

Known Asian wrongful conviction cases tend to be those involving particularly severe misconduct or high-profile official intervention, reflecting visibility and documentation biases rather than the full scope of wrongful convictions [15,29], which includes everyday, less-publicized cases. When significant segments of wrongful conviction data remain hidden, policymakers who rely on the most visible cases will form a distorted picture of the problem, shaped by the features of high-profile prosecutions. Systematic documentation of both high- and low-profile cases would correct the composition of what is known, and would reveal through aggregation that the scale of the problem is considerably larger than the headline cases alone suggest. Without a fuller picture, cross-border initiatives, such as unified training for forensic experts or joint review commissions, also lack the data-driven rationale needed to gain political support.

In the current article, we (1) summarize what is currently known about wrongful convictions in Asia; (2) review existing registries, innocence organizations, and documentation initiatives in Asia; and (3) present an elaborate operational map of the Asian Registry of Wrongful Convictions (ARWC): its rationale, scope, inclusion and exclusion criteria, development logic, documentation template, verification workflow, priority variables, and feasibility. The principal contribution of this paper is therefore infrastructural and methodological.

## 1 Contributing factors of wrongful convictions in Asia

Decades of research, particularly from North America and Europe, have repeatedly identified a relatively small number of recurring factors in known wrongful convictions, including false confessions, eyewitness misidentification, forensic and evidentiary errors, and forms of investigative or prosecutorial misconduct [12,[33], [34], [35]]. Although the volume of empirical work in Asia is smaller in comparison, available studies indicate that similar risks also appear across the region [14,15]. The factors discussed below are factors associated with known or officially documented wrongful convictions, not with the full hidden universe of wrongful convictions. Many other wrongful convictions may never become visible in registry-type data and may arise through mechanisms that are difficult to document.

**False Confession.** A significant factor contributing to wrongful convictions across countries are false confessions where individuals admit to crimes they did not commit due to individual vulnerabilities and/or situational pressures International studies identify several key mechanisms, including guilt-presumptive interrogation, prolonged questioning, sleep deprivation, repeated accusations, intimidation, deception (such as presenting false evidence), and the exploitation of suspect vulnerabilities such as youth or cognitive impairment [34,[36], [37], [38]]. Empirical studies from Asia reflect similar patterns that false confessions are repeatedly identified as a major contributing factor in documented cases and scholarship across multiple jurisdictions (See Le et al., 2024 for a systematic review). For example, research has indicated that interrogations in China often remain strongly guilt-presumptive and confession-oriented, with psychological pressure, inducements, and scripted questioning commonly reported during the investigative stage [29,[39], [40], [41]]. In Japan, drawing on case reviews and Ministry of Justice survey data on interrogation practice, scholars have highlighted how prolonged pre-trial detention, limited early access to legal counsel, and lengthy questioning can create conditions that heighten the risk of coerced or compliant confessions ([42,43]; see also [21,32]). Likewise, Taiwanese scholarship has documented the frequent occurrence of coerced confessions in wrongful conviction cases, particularly in capital and other high-profile prosecutions before the early 2000s [[44], [45], [46]].

**Eyewitness Misidentification.** Eyewitness misidentification is another well-established factor contributing to wrongful convictions across jurisdictions [35,47]. Decades of psychological research demonstrate that human memory is reconstructive and vulnerable to error, particularly under conditions commonly associated with criminal events, such as high stress, brief exposure, poor lighting, and the presence of a weapon [48]. Empirical research from Asia, though more limited in scope, reflects comparable risks. A recent systematic literature review of wrongful convictions in Asian countries identified eyewitness misidentification and false witness testimony as one of the recurring contributing factors across the Asian region, alongside false confessions, misconduct, and forensic errors [15]. The risk increases when identification evidence is treated as decisive even when it is internally inconsistent or when procedural protections are weak. For example, in China, scholars have described cases in which eyewitness accounts were relied upon despite contradictions, and where opportunities to test identification evidence, such as through effective cross-examination or safeguards against suggestion, were limited [29,49]. Studies from Singapore have also raised concerns about the reliability of eyewitness identification procedures and the limited use of scientifically informed safeguards during investigations [24]. Such errors also appear in Indonesia, as “suggestive lineup” tactics can nudge witnesses toward the wrong suspect [50].

**Forensic and Evidentiary Errors.** Forensic science can be a powerful ally of justice but also a dangerous liability when misapplied. Research from multiple jurisdictions has demonstrated that wrongful comvictions can occur when forensic evidence is improperly collected [51], inadequately validated [52], overstated in court [53], or interpreted through a lens of confirmation bias [54]. While DNA evidence is generally regarded as scientifically robust, research has shown that DNA results can nevertheless be misinterpreted or overstated in practice, particularly in cases involving complex analyses or probabilistic interpretation [52,55]. The available literature suggests that similar forensic and evidentiary vulnerabilities are also present in Asian criminal justice systems. Studies of wrongful conviction cases in China, for example, have identified forensic identification errors and overreliance on expert testimony as contributing factors in several wrongful convictions [29,39]. In Taiwan, at least one exoneration illustrates how a mixed Y-STR DNA profile was initially mischaracterized as compelling inculpatory evidence but was later revealed to be misleading [56]. Similar challenges have also been noted in parts of Southeast Asia, where studies have raised concerns about evidence handling practices and the capacity of forensic institutions to independently verify results [57,58].

**Investigatory and Prosecutorial Misconduct.** Research from multiple jurisdictions has shown that wrongful convictions may arise when investigators or prosecutors engage in practices such as suppressing exculpatory evidence, coercing or manipulating witnesses, presenting misleading forensic or testimonial evidence, or pursuing cases with excessive tunnel vision. In China, for example, research has shown that strict case-resolution deadlines and performance targets can foster bias and increase incriminating narratives at an early stage of the investigation [29]. In Japan, audio and video recording of interrogations covers only about 3% of criminal cases, and interrogations conducted outside physical detention are typically not recorded; as a result, interrogation practices and resulting statements may be difficult to verify or challenge [42]. While reviews of capital and other wrongful conviction cases in Taiwan have documented recurrent problems in the handling of physical evidence, such as loss, destruction, or contamination of exhibits by police and prosecutors [45,46], research from Indonesia similarly suggests that investigative practices may prioritize confessions over the systematic pursuit of alternative leads, and that weaknesses in disclosure obligations can limit the identification of exculpatory evidence [23,24]. Across contexts, such practices are often discussed as consequences of institutional incentives to resolve cases efficiently or maintain high conviction rates, rather than as isolated acts of individual wrongdoing [26,27].

**Limited or Inadequate Defense.** Limited or inadequate defense representation is another contributing factor associated with wrongful convictions across legal systems [33,59]. Research from China indicated that defendants, particularly those who are indigent or located in rural areas, may face limited access to effective legal representation, reducing opportunities to review evidence, consult experts, or challenge investigative practices [24,25]. For instance, in Vietnam, some exonerees noted receiving minimal attorney access prior to trial, thereby limiting access to thorough evidence review [15]. Scholars have also noted that professional and cultural norms within some legal systems may discourage defense lawyers from aggressively contesting official evidence, particularly in contexts where adversarial challenge to state authorities is institutionally constrained [25].

**Other Factors.** Perjury and informant testimony have also received sustained attention as important contributors to wrongful conviction [10,60]. The Asian literature also shows that informant testimony and witness perjury can contribute to wrongful convictions [15,61]. For example, in China, the Zhang Gaoping and Zhang Hui case involved false testimony from a fellow detainee, Yuan Lianfang, who gave coerced statements under police direction, showing how jailhouse-informant-type evidence can become highly prejudicial when it is not independently evaluated [15]. Beyond the procedural factors, structural features of the system itself can raise the risk of error: institutional pressure for case resolution, performance targets tied to conviction rates, limited recording of interrogations, and weak disclosure obligations have all been identified as conditions under which the factors above are more likely to arise; broader cultural, political, and media-related pressures can further amplify the risk of wrongful convictions [21,26,27,29].

## 2 Existing platforms addressing wrongful convictions in Asia

Despite the recurring nature of these contributing factors, systematic information about wrongful conviction cases, including how errors arise, how they are identified, and how they are ultimately addressed, remains fragmented, particularly in Asian jurisdictions. This gap makes it difficult to assess patterns, compare cases across jurisdictions, or translate lessons learned into meaningful legal and institutional reform. In response to similar challenges elsewhere, several regions have developed centralized wrongful conviction registries and platforms designed to document cases, synthesize evidence, and support research, advocacy, and policy development.

### 2.1 Global approaches

#### 2.1.1 Advocacy and post-conviction review

Wrongful conviction initiatives have taken a variety of institutional forms. In the United States, The Innocence Project (https://innocenceproject.org/), founded in 1992, pioneered the use of DNA testing in post-conviction litigation and has played a central role in exonerating wrongfully convicted individuals. Beyond individual cases, its work has contributed to reforms related to interrogation recording, forensic science standards, eyewitness identification procedures, and compensation for exonerees. Building on this model, the Innocence Network now connects dozens of organizations across multiple countries, facilitating knowledge exchange, training, and coordinated reform efforts.

A different institutional approach is exemplified by the Criminal Cases Review Commission (CCRC; https://ccrc.gov.uk/) in the England, Wales and Northern Ireland. Established under the Criminal Appeal Act 1995, the CCRC operates as an independent statutory body with the authority to investigate potential miscarriages of justice and refer cases back to appellate courts where there is a “real possibility” that a conviction will be quashed. Variants of this independent review model have since been adopted or adapted in other jurisdictions, including Norway and New Zealand. Canada is also moving toward a statutory independent review body modeled on the Criminal Cases Review Commission (CCRC) in the UK. In 2024, the Miscarriage of Justice Review Commission Act received royal assent, setting up a new independent commission in Winnipeg to review alleged wrongful convictions and make recommendations for re-trial or appeal.

In the Netherlands, the Knoops' Innocence Project (https://knoopsadvocaten.nl/en/innocence-project/) works on behalf of individuals claiming wrongful conviction, particularly by drafting review requests and pursuing re-examination before the Supreme Court or appellate courts where new evidence arises. Unusually, it operates from within a private law firm, a model that is uncommon but not unique (see Loevy & Loevy in the United States as a comparable example). This project is connected to the Innocence Network and assists in challenging convictions based on new forensic or testimonial material. Initiatives like Knoops' project function as national hubs for wrongful conviction advocacy, including supporting applications for review and contributing to broader debates about post-conviction safeguards. In addition, university-based initiatives also exist, such as the Netherlands' Project Gerede Twijfel and Belgium's KU Leuven's Voordeel van de Twijfel, which support case review through supervised student-led investigation.^2^ Moreover, professional judicial engagement also includes the Dutch Association for the Judiciary (Nederlandse Vereniging voor Rechtspraak; NVvR), the professional association of judges and public prosecutors, which describes its role as safeguarding the quality and accessibility of the judiciary.^3^

#### 2.1.2 Registries and documentation platforms

In addition to legal advocacy and case-review bodies, several initiatives focus specifically on documenting wrongful convictions through systematic data collection. The National Registry of Exonerations (NRE; https://exonerationregistry.org/) in the United States maintains a comprehensive, publicly accessible database of exoneration cases, providing detailed information on contributing factors, procedural histories, and case outcomes. By aggregating case-level data, the registry supports empirical research, policy evaluation, and public accountability. Canada has a dedicated registry documenting known wrongful conviction cases. Known as the Canadian Registry of Wrongful Convictions (https://www.wrongfulconvictions.ca/), this project compiles cases that have been overturned or otherwise recognized as miscarriages of justice, and makes them publicly visible as a research and advocacy resource. It is run by volunteer researchers and includes cases overturned by Canadian courts, providing data, timelines, and resources for understanding miscarriage patterns. This registry has been used by academics and advocates to highlight justice system failures, including wrongful guilty pleas and systemic obstacles to overturning wrongful convictions. The Miscarriages of Justice Registry at the University of Exeter (https://evidencebasedjustice.exeter.ac.uk/) documents cases in England and Wales from the past fifty years in which convictions were overturned because of problems with evidence or its evaluation.

The European Registry of Exonerations (EUREX; https://www.registryofexonerations.eu/) collects and standardizes information on exonerations across multiple European jurisdictions, currently documenting 144 exonerations from 20 countries [62]. Designed as a collaborative research infrastructure, EUREX accommodates several distinct legal systems, languages, and post-conviction procedures within one documentation framework, and enables cross-national comparison of wrongful conviction patterns and contributing factors.

### 2.2 Asian initiatives

#### 2.2.1 Advocacy and post-conviction review

Several initiatives in Asia have sought to address wrongful convictions primarily through legal advocacy and post-conviction review. The Taiwan Innocence Project (https://twinnocenceproject.org/en/home-en/), founded in 2012, provides pro bono legal assistance to individuals claiming wrongful conviction while also engaging in public education and policy advocacy. Its work has centered on post-conviction review in both DNA and non-DNA cases, using renewed forensic examinations, consultations with independent experts, and the introduction of revised scientific opinions in court to contest unsafe convictions, and on raising awareness of systemic issues such as eyewitness identification, forensic reliability, and interrogation practices within Taiwan's criminal justice system.

Similarly, the Innocence Project Japan (https://innocenceprojectjapan.org/en/home-2), established in the late 1990s, works to identify and challenge wrongful convictions through legal advocacy, scientific evidence, and public outreach. Operating within a criminal justice system characterized by strong prosecutorial dominance and historically low acquittal rates, the organization has contributed to public debate on coerced confessions, disclosure practices, recording of interrogations, and the need for more robust post-conviction review mechanisms in Japan.

#### 2.2.2 Registries and documentation platforms

In addition to advocacy-based initiatives, documentation-oriented efforts have begun to emerge. The China Wrongful Convictions Case Archive (https://usali.org/china-wrongful-convictions-case-archive), hosted by U.S.-Asia Law Institute, New York University, compiles information on documented wrongful conviction cases in China. By systematically collecting court decisions, case summaries, and secondary analyses, the archive provides an important research resource for examining patterns of error and post-conviction correction in a context where official data on wrongful convictions remain limited. Taken together, the jurisdictions reviewed here display a common asymmetry: advocacy and post-conviction review capacity exists and is growing, but documentation infrastructure remains minimal, and to our knowledge no jurisdiction in the region maintains a comprehensive public registry of corrected convictions.

## 3 Recommendations: establishing an Asian registry of wrongful convictions

The preceding sections show that wrongful convictions in Asia involve recurring procedural failures and structural conditions, yet information about such cases remains fragmented, inconsistent, and often inaccessible. While individual innocence projects, legal advocacy groups, and research initiatives exist, there is currently no regional infrastructure that systematically documents wrongful conviction cases across Asian jurisdictions in a consistent and transparent manner. This paper therefore proposes the development of an *Asian Registry of Wrongful Convictions (ARWC)* as a documentation-oriented platform designed to consolidate case information, support research, and facilitate cross-jurisdictional understanding. The inclusion framework presented here is a working framework under version control. Categories, markers, and thresholds will be refined as pilot cases surface configurations the current definitions handle poorly, with each change documented so that cases coded under earlier versions remain interpretable.

### 3.1 Purpose of the registry and design principles

ARWC will be conceived as an *academic and documentation-based* registry, and will not function as a body that determines guilt or innocence, reviews convictions, or provides legal representation. Its primary function will be to record and organize information on completed wrongful conviction cases based on official post-conviction actions, so that documented cases and their characteristics can be compared across the legal systems of the region. The registry is intended to complement, rather than replace, existing documentation-oriented initiatives. By providing a centralized documentation infrastructure, ARWC addresses a gap that limits comparative research, policy analysis, and evidence-based reform across the region.

A regional rather than national design was adopted for four reasons. First, in most Asian jurisdictions the number of officially corrected cases is small; separate national registries would each contain few cases and would be difficult to sustain. Second, documentation expertise, translation capacity, and comparative methodology can be pooled across jurisdictions, as EUREX demonstrates for Europe. Third, a shared framework makes cases comparable at the level of cases and characteristics, though not, for the reason discussed under interpretive limits below, at the level of counts as measures of prevalence. Fourth, a regional registry can seed later national registries rather than compete with them. The principal disadvantage is a heavier harmonization burden, since a regional registry must accommodate greater legal and linguistic diversity than a national one; the partial harmonization strategy described below is our response to the cost.

To demonstrate feasibility, key components of the registry have already been developed. These include a standardized documentation template, detailed scope definitions, and explicit inclusion and exclusion criteria. A draft website structure has also been prepared to illustrate how case information could be organized and accessed. These materials are intended as proof-of-concept tools and remain subject to refinement as the registry develops.

Four design principles guided the development of the registry. The first is verifiability: each included case must rest on traceable official post-conviction action, not merely on allegation or advocacy claim. The second is finality: the original conviction must already have become final under the ordinary procedures of the jurisdiction before the later corrective action occurred. The third is partial harmonization: the registry standardizes a core dataset for comparison while preserving local procedural labels, local-language terminology, and jurisdiction-specific context. The fourth is transparency about interpretive limits: ARWC documents officially recognized cases; it does not estimate prevalence, and its case counts should not be read as direct measures of how often wrongful convictions occur in a jurisdiction.

### 3.2 Scope and definition of wrongful conviction

For the purposes of ARWC, a wrongful conviction is defined as a criminal conviction that has been officially corrected or acknowledged as erroneous through post-conviction processes. Importantly, the registry does not independently assess innocence. Two scope boundaries follow from this definition. ARWC documents criminal convictions only: violent offences, non-violent offences, and juvenile convictions handled within criminal procedure all fall within scope, while administrative penalties and civil judgments do not. Convictions entered on an admission of guilt are within scope where the other criteria are met, whether through a guilty plea, a negotiated disposition, or a leniency mechanism conditioned on admitting guilt. Geographically, the registry is open to cases from any jurisdiction within the broadly defined Asian region, with the jurisdiction of each case recorded as descriptive data rather than fixed by a predetermined list.

Inclusion in the registry will be based on verifiable official actions taken after the conviction has become final under local law. ARWC uses finality as an operational stability requirement rather than as a universal legal concept, recognizing that “finality” carries different meanings across legal systems. For ARWC, a conviction is treated as final when ordinary appeals have been completed, the time for ordinary appeal has expired, or the judgment has otherwise entered legal effect under local law. ARWC does not require exhaustion of extraordinary remedies, such as retrial, post-conviction review, or special appeal, before a conviction is considered final.

This requirement is deliberately conservative: it ensures the registry documents completed convictions that were later corrected or acknowledged, rather than cases still moving through ordinary appellate review. Registries differ on this point. EUREX likewise requires finality; the NRE, the Canadian Registry of Wrongful Convictions, and the Miscarriages of Justice Registry for England and Wales do not, the last being built entirely on convictions overturned on appeal, and may therefore capture convictions corrected during ordinary appellate review, which ARWC excludes. Three considerations support the requirement for ARWC's purposes. First, in some Asian systems, such as China, the correction of error during ordinary appellate review is a routine function; including such cases would combine ordinary error-correction with post-finality correction and would count as wrongful convictions many cases that the local system does not treat as such. Second, finality provides a threshold that can be verified from official records across very different procedural systems. Third, finality stabilizes the record: a case entered after finality and official correction will not change status while proceedings continue. Because this design choice differs from some existing registries, case counts across registries will not be directly comparable.

Asian jurisdictions differ in how wrongful convictions are corrected or acknowledged. In some systems, error may be recognized through retrial acquittal or appellate reversal; in others, it may be reflected in prosecutorial admission of error, administrative rectification, compensation, or another official action that acknowledges the conviction was wrong or unreliable. ARWC therefore uses official post-conviction action, whether corrective or acknowledging, as its inclusion trigger; the three categories described below give this trigger operational form.

We adopt “wrongful conviction” rather than “exoneration” as the organizing term, following the Canadian Registry of Wrongful Convictions, which uses the same term and, like ARWC, treats official acknowledgment of error as sufficient for inclusion. The term suits this inclusion rule. “Exoneration” conventionally denotes formal clearing of the person, and would misdescribe entries admitted on the basis of a formal acknowledgment of error, such as a compensation decision expressly premised on wrongful conviction, or of innocence-related relief that leaves the conviction formally in place. “Wrongful conviction” instead names the object the registry tracks, the conviction and its correction, without presupposing the form that correction took. Substantively, however, the difference from exoneration-based registries is narrower than the change of vocabulary might suggest: like the NRE, ARWC requires an official post-conviction act indicating that the conviction should not stand, and most included cases will be ones those registries would also recognize. The differences lie in the conditions attached to that act: ARWC requires finality, which narrows its scope; ARWC does not require formal clearing of the person, as explained above, nor newly available evidence, and these omissions broaden it.^4^ The absence of a new-evidence requirement is taken up in the following section, where the inclusion trigger is defined.

### 3.3 How the inclusion framework was developed

The inclusion framework was not developed by simply copying a single Western registry definition. Rather, it emerged from a comparison of existing documentation projects and from iterative testing against pilot Asian materials.

As a starting point, we examined the selection logic used by the National Registry of Exonerations, EUREX, and the Canadian Registry of Wrongful Convictions. All three rely on post-conviction official action, but they were designed within different legal environments and use different thresholds or labels. Those models were therefore instructive but not fully transferable. For example, the NRE requires that the person be relieved of all consequences of the conviction, through a declaration of factual innocence, a pardon, an acquittal, or a dismissal, and that this occur after evidence of innocence not presented at the original trial became available; these specific instruments have no uniform counterpart across Asian systems, and several Asian post-finality corrections do not involve new evidence at all. In China, for example, the 2020 retrial acquittal of Zhang Yuhuan rested entirely on a re-examination of the original trial record: the court found that the physical evidence lacked demonstrated connection to the defendant and that his confessions were internally inconsistent, without any new fact, witness, or alternative suspect emerging [63]. The Canadian Registry is close to ARWC in its inclusion logic, extending to official acknowledgment of error including compensation decisions, but documents cases from a single national system. EUREX requires a final judgment followed by clearing (a declaration of innocence, acquittal, dismissal, or pardon), and treats new evidence as illustrative rather than mandatory; as a European registry, however, it does not encounter the same range of legal traditions, languages, and documentation practices found across Asia.

The next step was to test draft criteria against the procedural realities reflected in ARWC pilot materials and in the post-conviction pathways of jurisdictions that are already central to the project, including China, Indonesia, Japan, and Taiwan. This testing yielded design lessons directly. For example, a candidate case that initially appeared eligible proved on examination to have ended in an acquittal on ordinary appeal, so that no conviction had ever become final; this taught us to make finality confirmation the first screening step rather than a background assumption. In other materials, a compensation decision preceded, or substituted for, any judicial exoneration, which led us to treat formal acknowledgment of error as a distinct inclusion route rather than as a secondary feature of judicial reversal. Testing against these materials also revealed two recurring structural challenges. First, the same substantive correction of error may occur through very different procedural routes. For example, a final conviction may be corrected in China through trial supervision procedures, and in Indonesia through Peninjauan Kembali (PK), an extraordinary legal remedy; Table 1 lists who may initiate each route. Second, similar-sounding local remedies are not necessarily equivalent across legal systems. For example, a “retrial” in Japan (再審) and in China (再审) may both reopen a final conviction, but they differ in initiation, threshold, or legal consequence. The solution adopted here was therefore to classify cases by the function of the official post-conviction action rather than by the procedural name alone. In other words, the categories are functional rather than nominal.

**Table 1:** Illustrative post-conviction routes, official actions and ARWC categories.

Jurisdiction	Post-Conviction Route	Who May Initiate	Official Actions and ARWC Category
China	Retrial under the trial supervision procedure, following a petition, court or higher-court direction, or procuratorate protest	Party/legal representative/close relative (petition) or Court president/higher courts or Supreme People's Procuratorate or higher procuratorate	Retrial acquittal → judicial reversal (A). State compensation decision under the State Compensation Law, where the decision recognizes the conviction as wrongful → formal acknowledgment of error (B).
Indonesia	*Peninjauan Kembali* (PK; an extraordinary legal remedy) review of a final judgment	Terpidana (convicted person) or Heirs; if deceased: spouse, parents, child, or sibling or May be represented by specially-authorized advocate	PK vrijspraak (acquittal) or release from all charges → judicial reversal (A).
Japan	Retrial after final and binding judgment	The convicted person, prosecutor, representative, or eligible relative	Retrial acquittal → judicial reversal (A).
Taiwan	Retrial or Extraordinary appeal after final judgment	The convicted person, their statutory agent, a prosecutor, or their defense attorney acting in the interest of the defendant (Retrial); The Supreme Prosecutors Office via the Prosecutor-General (Extraordinary appeal)	Extraordinary-appeal acquittal → judicial reversal (A).

*Note.* Post-conviction routes and registry categories describe different things: a route is the procedure by which a final conviction is reopened, while a category records what the resulting official action accomplished. A single route may therefore produce different categories depending on its outcome, and some official actions recorded by the registry — administrative acknowledgments and compensation decisions among them — arise outside any reopening procedure. Local procedures are preserved as jurisdiction-specific data; the categories describe the function of the official action for inclusion purposes.

### 3.4 Developing inclusion/exclusion criteria across jurisdictions

As explained above, post-conviction remedies do not map neatly across jurisdictions. ARWC addresses this by using functional categories rather than assuming that identical procedural labels exist across systems. ARWC distinguishes between local post-conviction procedures and registry coding categories. Local procedures are recorded as jurisdiction-specific procedural fields. They are not themselves ARWC categories because they differ in legal threshold, who may initiate them, and what legal consequences they produce. The registry categories instead provide a functional coding layer that describes what the official action accomplished.

Before a case can be assessed for inclusion, the contributor must first confirm that the original conviction had become final under the jurisdiction's normal procedures, applying the definition of finality set out above. The inclusion trigger is the official corrective action itself, whatever prompted it: ARWC does not require that new evidence have emerged. The NRE, the Canadian Registry of Wrongful Convictions, and the Miscarriages of Justice Registry for England and Wales all require some form of newly available evidence, and the Canadian Registry expressly excludes convictions overturned without it. This choice reflects regional practice: in some Asian systems, as the Chinese example above illustrates, a final conviction may be corrected on the ground that the original evidence was insufficient, without any new evidence emerging.

Once finality is established, ARWC applies three categories of official post-conviction action:

Category A: Judicial reversal. These are cases in which a court later ends, removes, or nullifies the finding of guilt after the conviction became final. This category includes retrial acquittals, extraordinary appeal decisions quashing a final judgment, convictions vacated or annulled, and comparable judicial acts that formally undo the conviction.

Category B: Formal acknowledgments of error. This category captures cases in which a public authority officially recognizes that the conviction was wrong even when no judicial reversal has occurred. Prosecutorial admissions, compensation awards that explicitly recognize wrongful conviction, administrative rectification decisions, and equivalent formal acts fall into this category. What distinguishes Category B is that the official instrument itself speaks to the validity of the conviction.

Category C: Innocence-related relief or equivalent remedy. This category covers cases in which official relief is granted to the individual and the surrounding official documentation ties that relief to wrongful conviction concerns, although the instrument granting relief does not itself address the validity of the conviction. Pardons, commutations, and comparable mechanisms may fall here when the available documentation shows that the relief followed from innocence concerns rather than from discretionary mercy alone. What distinguishes Category C from Category B is therefore where the innocence-related statement appears: in the instrument itself, or only in the record surrounding it.

Three examples illustrate how the categories operate. A retrial acquittal that removes a final conviction is a judicial reversal: a court has ended the finding of guilt. A state compensation decision stating that a conviction was wrongful is a formal acknowledgment of error, because the document itself pronounces on the conviction even though the conviction may still stand. A pardon whose text grants release without addressing guilt, issued where official documentation elsewhere records serious doubts about the conviction, is innocence-related relief, because the innocence link is established by the surrounding record rather than by the instrument. Taken together, the three categories are intended to be exhaustive, so that any verifiable official post-conviction action falls within at least one of them, whatever procedural form it takes locally; the boundary between Categories B and C affects how a case is described rather than whether it is included.^5^ Table 1 illustrates how these categories map onto the post-conviction routes of four jurisdictions.

A single case may involve more than one official action, and the categories are recorded as non-exclusive checkboxes: a case is marked for each category that applies. For example, where a retrial acquittal is followed by a state compensation decision expressly acknowledging the wrongful conviction, the case is marked under both judicial reversal and formal acknowledgment of error. The registry does not designate one category as primary, because the categories function as inclusion criteria rather than as an analytic variable: what the registry counts is cases, each counted once upon inclusion. Where descriptive tallies by category are reported, they are non-exclusive and labeled as such. The sequence of official actions in a case is preserved independently, in a dated list recording each action's instrument, issuing authority, and procedural route, so that how recognition of error developed over time remains visible without being forced into a ranking.

Cases are *excluded* where: i) proceedings are ongoing or appeals are unresolved; ii) claims of wrongful conviction rely solely on personal assertions without official recognition or credible documentation; iii) the outcome reflects discretionary leniency without acknowledgment of error. This approach is informed by existing international registries (e.g., NRE) but adapted to ARWC's cross-jurisdictional documentation purpose.

### 3.5 Worked examples of inclusion decisions

Table 2 applies these criteria to a set of representative scenarios, including cases that fall outside the registry. The exclusions are as instructive as the inclusions: an acquittal on ordinary appeal is excluded because no final conviction ever existed, and a pardon granted on humanitarian grounds alone is excluded because relief without a documented link to wrongful-conviction concerns does not establish that the conviction was in error.

**Table 2:** Worked examples of ARWC inclusion logic.

Example	Include?	Category	Reason
Sample case (Supplement B)	Yes	Category A (Judicial reversal); Category B (Formal acknowledgments of error)	The conviction was reversed by a retrial acquittal, and a later compensation decision formally acknowledged the wrongful conviction.
Final conviction acquitted on extraordinary review (Peninjauan Kembali) after another person confesses to the crime	Yes	Category A (Judicial reversal)	The court later removed the legal finding of guilt through a post-conviction judicial process.
Compensation decision explicitly recognizing wrongful conviction, but no retrial acquittal yet	Yes, if documentation is sufficient	Category B (Formal acknowledgments of error)	A competent public authority formally acknowledged error even without a full judicial exoneration.
Executive pardon granted for humanitarian reasons only	No	Not applicable	Relief alone is insufficient unless the documentation ties the action to wrongful-conviction concerns.
Acquittal on ordinary appeal before conviction became final	No	Not applicable	The case fails the finality requirement because the error was corrected before a final conviction existed.

### 3.6 Documentation structure and harmonization strategy

ARWC uses a structured case documentation template designed to capture details including: age at conviction; jurisdiction and court level; offense and sentence; types of evidence relied upon at trial; identified contributing factors (e.g., confession evidence, forensic issues); post-conviction outcome and mechanism; other information (e.g., actual perpetrator identified, subsequent victims); supporting documentation (Table 3 illustrates this standardization logic across five representative dimensions; Supplement A gives the complete field-by-field specification). In addition to standardized fields, the template includes free-text fields that allow contributors to describe procedural context, legal terminology, and institutional features specific to each jurisdiction.

**Table 3:** Standardized fields and preserved legal difference.

Dimension	Standardized across cases	Preserved as local detail
Official action	Category A (Judicial reversal)	Local procedural route and issuing authority
	Category B (Formal acknowledgments of error)	
	Category C (Innocence-related relief or equivalent remedy)	
Offense	Broad offense type for comparison	Offense name as stated in the original language
Procedure	Finality confirmation and correction stage	Jurisdiction-specific legal mechanism
Evidence and factors	Standard check-box style coding plus narrative summary	Jurisdiction-specific explanation of how the factor operated
Sources	Source type and date	Original citation and document title

*Note.* Partial harmonization is best understood as a middle path between two extremes. Pure local description without standardized fields would make comparison very difficult. Full standardization that erased local meaning would distort the data. ARWC instead standardizes where necessary for comparison and preserves local legal nuance where necessary for accuracy.

The critical methodological issue is how to use standardized fields without erasing legal differences across jurisdictions. ARWC addresses this through a partial harmonization approach. Core data fields are standardized to support basic comparison, but local procedural names, local-language legal terms, and jurisdiction-specific narrative context are preserved rather than overwritten (Supplement B), because English labels necessarily approximate local procedures rather than replicate their exact legal meaning; preserving the original term allows that gap to be checked rather than assumed away. The registry, therefore, does not force all jurisdictions into one supposedly universal vocabulary of wrongful conviction. This design choice allows the registry to accommodate legal diversity across Asia while still enabling comparative analysis. Differences across systems are treated as analytically meaningful data rather than inconsistencies to be corrected.

### 3.7 Submission and preliminary verification

ARWC is designed to have an open but screened submission system allowing contributions from, for example, researchers, legal practitioners, journalists, innocence organizations, and civil society actors. Submissions must be accompanied by at least one traceable official document or equivalent authoritative record.

A workable preliminary workflow is as follows. First, the coordinating team receives the submission and checks whether the conviction had become final under local law. Second, the team verifies that the claimed official action fits one of the inclusion categories and is supported by the submitted documentation. Third, if the case passes that threshold, a case ID is assigned, and the contributor completes the core metadata fields. Fourth, the coordinating team prepares the structured English summary and codes the standardized fields. Fifth, the contributor or another qualified jurisdictional reviewer confirms that the English summary accurately reflects the original-language materials. Sixth, a publication decision is made, and the case is released with its status category and source information visible. English was selected as the registry's working language for two reasons: it is the shared language of international comparative research, and, precisely because it is not the official language of any covered jurisdiction, it privileges no legal system's terminology over another's. Original-language legal terms, procedural names, and case names are preserved in dedicated fields alongside the English summaries, consistent with the partial harmonization approach.

Where cases are ambiguous, especially with respect to finality, the meaning of the official action, or the relationship between multiple post-conviction acts, the case will be reviewed by at least two team members and logged in a brief coding memorandum. In later phases of the registry, formal inter-rater agreement checks and a version-controlled codebook would further improve consistency.

### 3.8 Database architecture and field structure

ARWC is designed as a structured database rather than a collection of narrative case summaries. Several fields will be stored as structured variables to support searching, filtering, and later analysis. Dates will be recorded using the most precise information available, with separate fields for year, month, and day where available, and a date-precision flag where only the year is known. “Years lost” will be stored as a numeric field, with an approximation flag where the value is estimated from available records. Compensation will be recorded as a separate field, including whether compensation was awarded, the amount, currency, year, issuing authority, and whether the compensation decision itself functioned as an acknowledgment of error. Each official action also records the basis stated in the decision, using a controlled vocabulary that distinguishes corrections resting on the insufficiency of the original evidence from those resting on new evidence of innocence or on procedural or legal error.

Jurisdiction-level information, such as country or region, legal-system family, and general procedural-system type, will be stored separately from case-level information and linked to individual case entries. This avoids unnecessary repetition and allows jurisdictional descriptions to be updated over time without altering the historical coding of each case. Case-level variables will include the offense, sentence, court level, evidence relied upon, contributing factors, official action, key triggers, and source documents. Where relevant and ethically appropriate, the registry may also include structured victim-related fields, such as number of victims, victim age group, victim-defendant relationship, or whether the alleged victim later reappeared. These fields will be used only when they are available in public or submitted documentation and can be recorded without unnecessary disclosure of sensitive personal information.

Contributing factors and key triggers will use structured multi-select fields accompanied by optional free-text explanation. Contributing factors are recorded at two levels: a parent factor, required where the factor applies, and an optional subcategory recorded where the documentation supports it. For example, “Torture or unlawful interrogation” is coded as a subcategory of confession-related factors and cross-coded to investigative misconduct. The structural conditions discussed above are recorded as a contributing factor, but will not be treated as a permanent label attached to a jurisdiction. Instead, it will be coded at the case level where the documentation indicates that a structural feature, such as disclosure rules, interrogation recording practices, or institutional pressure for case closure, was relevant at the time of investigation, conviction, or correction. This allows ARWC to capture historically specific conditions without implying that a jurisdiction's legal system is static. The registry will present case information in three layers: a filterable summary view, a full case entry, and a downloadable structured dataset for research use. Supplement B shows the complete record; fields marked internal in Supplement A appear in none of the three layers, or only in aggregated form.

### 3.9 Feasibility, governance, and long-term sustainability

The feasibility of ARWC depends on conceiving it as a phased documentation infrastructure. While a meaningful pilot registry does not require the immediate capacity to investigate every potential case in every Asian jurisdiction, it does need a clear codebook, a modest web platform, source management, translation and verification capacity, and regional contributors with jurisdiction-specific expertise. In practical terms, early support would likely be needed for website hosting and maintenance, student or research assistant time, translation support where necessary, legal-procedural consultation, and data management. A realistic early phase would therefore begin with jurisdictions in which contributors, source materials, and verification capacity are already available, and then expand as the contributor network and codebook mature.

A plausible governance structure is a coordinating team responsible for registry-wide standards and publication decisions, paired with regional co-developers or contributors who identify cases and help verify local-language materials. This distributed structure is particularly important for a registry covering multiple legal systems and languages. It reduces the risk of centralizing interpretive authority in one institution that lacks the relevant local expertise, and it helps ensure that case entries remain grounded in jurisdiction-specific knowledge.

Political sensitivity is a real but manageable issue. The risk for a wrongful conviction registry to be perceived as accusatory or politically inconvenient is reduced when the registry is methodologically restrained. ARWC documents cases only where the system itself has already taken official action to correct or acknowledge error. It is therefore a secondary aggregation platform. Even so, long-term sustainability will depend on careful governance, accurate case framing, stable institutional hosting, and partnerships that do not depend entirely on any single individual.

### 3.10 Interpretive limits and responsible use

A registry of officially recognized wrongful convictions can be extremely valuable, but it has clear interpretive limits. The most important is that registry counts are not prevalence estimates. Documented cases are the visible portion of a larger population of wrongful convictions, most of which never generate the official corrective action that would make them countable at all; the registry can therefore describe only what has surfaced. A second and distinct issue concerns comparison among the cases that do surface: A jurisdiction with more documented cases may have more wrongful convictions, but it may also have more transparent correction mechanisms, more accessible public records, stronger contributor networks, or more active post-conviction review processes. Conversely, a jurisdiction with few documented cases may not have fewer wrongful convictions; it may simply have weaker documentation or fewer official correction pathways.

ARWC is best understood as a record of officially documented corrective actions, not as a ranking device and not as a direct measure of comparative innocence or guilt across countries. Absence of cases in the registry should therefore be interpreted as absence of captured, verifiable, officially corrected cases under the registry criteria at a given point in time, not as evidence that wrongful convictions do not occur there. The registry captures only known, officially documented cases; many miscarriages of justice likely remain unknown and uncorrected, and some arise through pathways not emphasized in this paper, such as plea bargaining or pre-trial detention.

## 4 Conclusion

The reality of wrongful convictions in Asia serves as a stark reminder of the fallibility inherent in even the most efficient criminal justice systems. As this paper has demonstrated, wrongful convictions across the region are not merely isolated incidents of bad luck; documented cases across the region repeatedly reveal the same systemic vulnerabilities, ranging from an overreliance on coerced confessions and flawed forensic evidence to the immense institutional pressures for case closure. These errors do more than just punish the innocent; they inflict deep social and psychological harm on individuals [64] and fundamentally erode the public's trust in the rule of law [65].

While Western jurisdictions have long used centralized documentation to drive reform, the Asian context remains uniquely challenged by fragmented data, language barriers, and a lack of cross-border transparency. The proposed Asian Wrongful Convictions Registry is a practical response to the fragmentation of wrongful conviction information across the region. ARWC may also help reduce the under-representation of some Asian countries. By documenting cases based on official post-conviction actions, preserving legal diversity, and providing a transparent structure for data collection and verification, ARWC offers a foundation for systematic research, collaboration, and policy discussion. As an evolving documentation infrastructure, it represents a concrete first step toward making wrongful convictions in Asia more visible, comparable, and better understood.

## CRediT authorship contribution statement

**Yiwen Zhang:** Writing – review & editing, Writing – original draft, Methodology, Investigation, Conceptualization, Project administration. **Henry Otgaar:** Writing – review & editing, Writing – original draft, Supervision, Methodology, Investigation, Conceptualization. **Yikang Zhang:** Writing – original draft, Methodology, Investigation, Conceptualization. **Nathanael E.J. Sumampouw:** Methodology, Investigation, Conceptualization. **Tsania Isa:** Writing – original draft, Methodology, Investigation, Conceptualization. **Jianqing Wang:** Methodology, Investigation, Conceptualization. **Yoshiyuki Nishi:** Writing – original draft, Methodology. **Yun-Ching Ko:** Methodology, Writing – review & editing.

## Declaration of AI usage

During the revision of this manuscript, the authors used ChatGPT and Claude to assist with language refinement and organization of revision materials. All substantive arguments, citations, interpretations, and final wording were reviewed, verified, and approved by the authors. The authors take full responsibility for the final manuscript.

## Declaration of competing interests

The authors declare that they have no known competing financial interests or personal relationships that could have appeared to influence the work reported in this paper.

## Footnotes

1 In this paper, exoneration and related post-conviction outcomes are treated as verifiable official actions that can be used as inclusion triggers for documentation, not as the focus of analysis.

## Footnotes

2 See Project Gerede Twijfel (ALLP/VU Amsterdam): https://allp.nl/nl/projectgeredetwijfel/and Voordeel van de Twijfel (KU Leuven): https://www.kuleuven.be/voordeelvandetwijfel.

3 See Nederlandse Vereniging voor Rechtspraak: https://www.nvvr.org/adviezen/adviescommissie-afgesloten-strafzaken-acas/.

4 The inclusion criteria, the conditions a case must satisfy to enter each registry, are, in summary: The NRE requires that the person have been relieved of all consequences of the conviction and either declared factually innocent or granted a complete pardon, acquittal, or dismissal, following post-conviction re-examination, with evidence of innocence that was not presented at trial. EUREX requires conviction by final judgment followed by official clearing (declaration of factual innocence, acquittal, dismissal of all charges, or complete pardon); EUREX describes new evidence as an illustrative rather than a mandatory basis for clearing. The Canadian Registry of Wrongful Convictions requires that a conviction be overturned based on new matters of significance related to guilt, and generally excludes convictions overturned without new evidence; it additionally includes cases in which a state official, through a free pardon or other decision including the payment of compensation, recognizes that a person ought not to have been convicted. The Miscarriages of Justice Registry (England and Wales) requires that a guilty verdict have been overturned on appeal on the basis of fresh evidence and factual error at trial, with no substituted conviction, at least one year between conviction and successful appeal, and, where a retrial occurred, an acquittal at that retrial. Criteria are summarized from each registry's published inclusion statement; readers should consult the originals for the full text.

5 Both categories cover ground that the National Registry of Exonerations addresses within its single exoneration category, through innocence-based pardons and declarations of factual innocence. ARWC records the distinction because the two forms of official action are not equivalent as documentation: an authority that grants relief without pronouncing on the conviction has done something different from one that formally acknowledges error. The categories are defined by the range of official actions the region's legal systems can produce rather than by the composition of the current case set; no pilot case has yet been coded under Category C.
