# Supplementary material for: Towards an Asian registry of wrongful convictions
Source: Forensic Sci Int Synerg. 2026 Sep 2;13:100726. doi: 10.1016/j.fsisyn.2026.100726 (PMC13564123; doi:10.1016/j.fsisyn.2026.100726)
Supplement: Supplementary file 1 [file mmc1.docx]

**Supplement A. ARWC Data Dictionary (Codebook Version 1.0 — Working Framework Under Version Control)**

This data dictionary specifies every field in an ARWC case record: the field name, its data type, the permitted values where a controlled vocabulary applies, and whether the field is published or internal. Supplement B illustrates the coding of a single case using these fields. In Supplement B, bold entries are field names and the adjacent column contains the values entered.

Data types: CV = controlled vocabulary (permitted values listed); Num = numeric; Date = structured date (separate year, month, day, with a precision flag recording whether the day, month, or only the year is known); Y/N = yes/no; Y/N/U = yes/no/unknown; Text = free text; ID = assigned identifier; Link = reference to another record; Rep = repeating group of fields.

Visibility: Public fields appear in the published case entry. Internal fields are retained in the registry's records and appear publicly only in aggregated or redacted form.

*The framework is maintained under version control: categories, markers, and controlled vocabularies will be refined as pilot cases surface configurations the current definitions handle poorly, with each change documented so that cases coded under earlier versions remain interpretable. Controlled vocabularies below are initial sets and are extendable through codebook versioning.*

**0. Jurisdiction Record (stored separately; linked to case records)**

Jurisdiction-level information is stored once per jurisdiction and linked from each case entry, so that jurisdictional descriptions can be updated over time without altering the historical coding of individual cases. Legal-system family and procedural-system type are proposed by contributors and confirmed by a co-developer or contributor with expertise in that jurisdiction. These are contested classifications on which comparatists differ, and the registry does not assign them centrally: doing so would place interpretive authority over the legal systems it documents with a coordinating team that lacks standing in most of them. The classification is recorded together with the role and date of those who proposed and confirmed it; individual identities are held internally, consistent with the treatment of contributor identity.

| **Field** | **Type** | **Permitted values / format and notes** | **Visibility** |
| --- | --- | --- | --- |
| Jurisdiction ID | ID | Assigned; format JUR-[code]-## | Public |
| Country / Region | Text | As identified by the contributor | Public |
| Legal System Family | CV | Civil law; common law; mixed; religious-law components; other (contributor-provided) | Public |
| Procedural System Type | CV | Inquisitorial; adversarial; mixed; other (contributor-provided) | Public |
| Finality Rules Summary | Text | Brief description of how convictions become final in this jurisdiction; general legal background lives here, not in case entries | Public |
| Post-Conviction Routes Summary | Text | Local post-conviction procedures with original-language names and English glosses | Public |
| Classification Source | Text | Role and date of those who proposed and confirmed the legal-system family and procedural-system type (e.g., “contributor-proposed, confirmed by jurisdictional co-developer, 2026”); individual identities are recorded internally | Public |

**A. Case Identification**

| **Field** | **Type** | **Permitted values / format and notes** | **Visibility** |
| --- | --- | --- | --- |
| Case ID | ID | Format ARWC-[ISO country code]-### (e.g., ARWC-CHN-001) | Public |
| Jurisdiction ID | Link | Links to the jurisdiction record (Section 0) | Public |
| Case Name (Original Language) | Text | In the original script | Public |
| Original Language | CV | Language name (e.g., Chinese (Simplified); Japanese; Indonesian) | Public |
| Case Name (English) | Text | Working English name | Public |
| Entry Created / Last Updated | Date | Structured dates; codebook version used at entry recorded | Public |
| Contributor Identity | Text | Name and affiliation of the submitting contributor | Internal |

**B. Individual**

| **Field** | **Type** | **Permitted values / format and notes** | **Visibility** |
| --- | --- | --- | --- |
| Individual Name (Original Language) | Text | In the original script | Public |
| Individual Name (Romanized / English) | Text |  | Public |
| Gender | CV | Male; female; other; unknown | Public |
| Age at Conviction | Num | Integer, years | Public |
| Executed | Y/N | If Yes, Date of Execution is required | Public |
| Date of Execution | Date | Structured date with precision flag; recorded here, never inside Years Lost | Public |
| Years Lost | Num | Years of liberty deprivation attributable to the conviction, from detention to release or, where the person was executed, to execution; decimal permitted | Public |
| Years Lost: Approximation Flag | Y/N | Yes where the value is estimated from available records | Public |
| Posthumous Case | Y/N | Yes where the corrective action occurred after the individual's death | Public |

**C. Offense, Sentence, and Victim-Related Fields**

| **Field** | **Type** | **Permitted values / format and notes** | **Visibility** |
| --- | --- | --- | --- |
| Offense Category | CV (multi-select) | Homicide; sexual offense; robbery; assault; kidnapping or abduction; arson; drug offense; fraud or property offense; public-order offense; other (specify). English controlled vocabulary for comparison; initial set, extendable | Public |
| Offense as Charged (Original Language) | Text | Charge as stated in the original language | Public |
| Offense as Charged (English Gloss) | Text | English rendering of the local offense name | Public |
| Sentence | CV + detail | Death (immediate); death with reprieve; life imprisonment; fixed term (years, Num); other (specify) | Public |
| Court of Original Trial | Text | Name, original language and English | Public |
| Evidence Relied Upon at Trial | CV (multi-select) | Confession; eyewitness identification; forensic evidence (specify type); documentary evidence; informant or witness testimony; circumstantial evidence; other (specify) | Public |
| Number of Victims | Num | Integer; as identified in the case documents | Public |
| Victim Identifying Details | Text | Names or other identifying information | Internal |
| Victim Age Group | CV | Minor; adult; mixed; unknown | Public |
| Victim–Defendant Relationship | CV | Stranger; acquaintance; family member; other; unknown | Public |
| Alleged Victim Later Reappeared | Y/N/U | For no-crime cases in which the presumed victim was found alive | Public |
| Actual Perpetrator Identified | Y/N/U |  | Public |
| Subsequent Victims Attributed to Actual Perpetrator | Y/N/U | With optional non-identifying note | Public |
| Victim-Related Note | Text | Non-identifying context only | Public |

**D. Finality**

| **Field** | **Type** | **Permitted values / format and notes** | **Visibility** |
| --- | --- | --- | --- |
| Finality Basis | CV | Ordinary appeals completed (second-instance or appellate judgment issued); ordinary appeal period expired; judgment otherwise entered into legal effect under local law | Public |
| Date of Finality | Date | Structured date with precision flag | Public |
| Finality Note | Text | At most two sentences; case-specific facts only | Public |

**E. Official Post-Conviction Action**

The three categories are recorded as non-exclusive checkboxes: a case is marked for each category that applies. No category is designated primary; the registry counts cases, and any descriptive tallies by category are non-exclusive and labeled as such. The sequence of official actions is preserved in the dated Official Actions list.

*The categories are defined by the range of official actions the region’s legal systems can produce rather than by the composition of the current case set. No pilot case has yet been coded under Category C.*

| **Field** | **Type** | **Permitted values / format and notes** | **Visibility** |
| --- | --- | --- | --- |
| Category: Judicial Reversal (A) | Checkbox | A court ended, removed, or nullified the finding of guilt after finality, in whole or in part | Public |
| Category: Formal Acknowledgment of Error (B) | Checkbox | A public authority's instrument itself speaks to the validity of the conviction (e.g., compensation decision expressly premised on wrongful conviction; prosecutorial admission; administrative rectification) | Public |
| Category: Innocence-Related Relief (C) | Checkbox | Official relief granted to the individual where the surrounding official documentation ties the relief to wrongful-conviction concerns, although the instrument granting relief does not itself address the validity of the conviction | Public |
| Official Actions List | Rep | One entry per official action; fields below | Public |
| — Action Type | CV | Retrial acquittal; post-finality appellate acquittal; conviction vacated or annulled; extraordinary appeal decision quashing a final judgment; prosecutorial admission of error; state compensation decision; administrative rectification; pardon (innocence-linked); commutation (innocence-linked); other (specify) | Public |
| — Category | CV | A; B; C (per the definitions above) | Public |
| — Document Type | Text | Original language and English | Public |
| — Date of Action | Date | Structured date with precision flag | Public |
| — Issuing Authority | Text | Original language and English | Public |
| — Procedural Route | Text | Original language and English | Public |
| — Basis Stated in the Decision | CV | Insufficiency of the original evidence; new evidence of innocence; procedural or legal error; other (specify); not stated | Public |
| — Summary | Text | One to two sentences | Public |
| Compensation Awarded | Y/N/U |  | Public |
| — Amount / Currency / Year | Num / CV / Num | Currency as ISO code or standard abbreviation | Public |
| — Issuing Authority | Text |  | Public |
| — Decision Acknowledges Wrongful Conviction | Y/N | If Yes, the decision is typically also recorded as a Category B official action | Public |

**F. Case Summary**

Free text under three fixed headings, identical for every case: (1) Background and Original Conviction; (2) Emergence of Grounds for Correction; (3) Path to Official Action. Recommended length 150–300 words per heading. Public.

**G. Contributing Factors**

Controlled multi-select. The parent factor is the coding unit and is required where the factor applies; the subcategory is an optional refinement, recorded only where documentation supports it, so that thin documentation does not force either guessing or non-coding. Each selected factor is accompanied by a short factual note stating its documentary basis (what the judgment, decision, or record shows). Notes record facts, not coding justifications. Public.

| **Factor and subcategories** | **Coding** | **Optional note** | **Notes on use** |
| --- | --- | --- | --- |
| 1. False or coerced confession | Checkbox | Free text | Confession evidence contributed to the conviction and was retracted, excluded, or found unreliable |
| 1a. Torture or physical coercion | Checkbox | Free text | Where selected, cross-code to factor 4 |
| 1b. Prolonged detention or questioning without counsel | Checkbox | Free text |  |
| 1c. Deception, inducement, or threats | Checkbox | Free text |  |
| 2. Eyewitness misidentification | Checkbox | Free text |  |
| 2a. Suggestive identification procedure | Checkbox | Free text |  |
| 2b. Identification treated as decisive despite inconsistency or weak conditions | Checkbox | Free text |  |
| 3. Forensic or evidentiary error | Checkbox | Free text | Includes misapplied, overstated, or invalid forensic evidence |
| 4. Investigative or prosecutorial misconduct | Checkbox | Free text |  |
| 4a. Suppression of exculpatory evidence | Checkbox | Free text |  |
| 4b. Failure to act on exculpatory developments | Checkbox | Free text | E.g., declining to pursue a confession by another person |
| 4c. Fabrication of evidence | Checkbox | Free text |  |
| 5. Inadequate defense | Checkbox | Free text |  |
| 6. Perjury or false accusation (including informant testimony) | Checkbox | Free text |  |
| 7. Systemic or structural issue | Checkbox | Free text (specification required) | Coded at case level where documentation shows a structural feature (e.g., disclosure rules, interrogation-recording practices, institutional pressure for case closure) was relevant at the time of investigation, conviction, or correction; not a permanent jurisdiction label |
| 8. Other contributing factor | Checkbox | Free text (specification required) | Recurring specifications will be reviewed for promotion to named factors in later codebook versions |

**H. Key Triggers of Official Action**

Controlled multi-select recording what prompted the official action. Triggers record the impetus for a correction, not its legal basis or its procedural route, both of which are recorded in Section E. As with contributing factors, each value is a checkbox with an optional free-text note. Public.

| **Trigger** | **Coding** | **Optional note** | **Notes on use** |
| --- | --- | --- | --- |
| Real perpetrator identified | Checkbox | Free text | Another person identified as responsible, whether or not charged |
| Confession by another person | Checkbox | Free text | May co-occur with the above; recorded separately because a confession may not lead to identification or charge |
| New or re-examined forensic evidence | Checkbox | Free text | Includes re-testing of existing material |
| Recantation of testimony | Checkbox | Free text |  |
| Newly disclosed or discovered evidence | Checkbox | Free text | Evidence not before the original court, including material previously withheld |
| Sustained petitioning or family advocacy | Checkbox | Free text |  |
| Involvement of an innocence organization or counsel | Checkbox | Free text |  |
| Media or public attention | Checkbox | Free text |  |
| Official review initiative | Checkbox | Free text | Court-, procuratorate-, or commission-initiated review; records the impetus for the action, not the procedural route, which is recorded in Section E |
| Other | Checkbox | Free text (specification required) |  |

**I. Inclusion Verification and Coding Memorandum**

The verification table is published with each case in simplified form, showing why the case satisfies the registry criteria. Category rows are marked for each applicable category; no category is designated primary. The coding memorandum is internal.

| **Field** | **Type** | **Permitted values / format and notes** | **Visibility** |
| --- | --- | --- | --- |
| Criminal offence | CV | PASS; FAIL | Public |
| Finality | CV | PASS; FAIL; with basis recorded in Section D | Public |
| Official post-conviction action | CV | PASS; FAIL | Public |
| Judicial Reversal (A) | CV | MARKED; NOT MARKED | Public |
| Formal Acknowledgment of Error (B) | CV | MARKED; NOT MARKED | Public |
| Innocence-Related Relief (C) | CV | MARKED; NOT MARKED | Public |
| Documentation | CV | PASS; FAIL; at least one traceable official document required | Public |
| Basis (per criterion) | Text | Short statement of the documentary basis for each determination | Public |
| Inclusion determination | CV | INCLUDED; EXCLUDED; PENDING | Public |
| Coding memorandum | Text | Reviewer notes on ambiguous points; two-reviewer log where the case was referred | Internal |
| Codebook version at coding | Text | Version of the data dictionary applied | Public |

**J. Supporting Documentation and Sources**

| **Field** | **Type** | **Permitted values / format and notes** | **Visibility** |
| --- | --- | --- | --- |
| Source entries | Rep | One entry per source; fields below; at least one traceable official document is required for inclusion | Public |
| — Source Type | CV | Judgment; compensation decision; prosecutorial document; administrative document; official gazette entry; legislation; academic publication; media report (supporting context only, never the sole basis for inclusion) | Public |
| — Title (Original Language) | Text |  | Public |
| — Title (English) | Text |  | Public |
| — Issuing Authority / Author | Text |  | Public |
| — Date | Date | Structured date with precision flag | Public |
| — Availability Note | Text | E.g., published in official gazette; on file with contributor | Public |

**Supplement B. Sample Case Entry**

This entry represents the full back-end record. Fields marked Internal in Supplement A are shown here for illustration and flagged; in the public display they appear only in aggregated or redacted form. Bold entries are field names; the adjacent column contains the values entered.

**Linked Jurisdiction Record**

| **Jurisdiction ID** | JUR-CHN-01 |
| --- | --- |
| **Country / Region** | China — Hebei Province (中国河北省) |
| **Legal System Family** | Civil law (continental) (大陆法系) [contributor-provided] |
| **Procedural System Type** | Inquisitorial with limited adversarial elements [contributor-provided] |
| **Finality Rules Summary** | Two-instance final-judgment system (两审终审制): a conviction becomes legally effective upon issuance of the second-instance judgment, or upon expiry of the appeal period where no appeal is filed. |
| **Post-Conviction Routes Summary** | Retrial (再审) under the trial supervision procedure (审判监督程序), initiated by petition, court, or procuratorate protest; state compensation under the State Compensation Law (国家赔偿法). |

**A. Case Identification**

| **Case ID** | ARWC-CHN-001 |
| --- | --- |
| **Jurisdiction ID** | JUR-CHN-01 |
| **Case Name (Original Language)** | 聂树斌故意杀人、强奸妇女案 |
| **Original Language** | Chinese (Simplified) |
| **Case Name (English)** | Nie Shubin intentional homicide and rape case |
| **Entry Created / Last Updated** | 2026 / 2026 (Codebook v1.0) |
| **Contributor Identity** | Prepared by the authors for illustrative purposes |

**B. Individual**

| **Individual Name (Original Language)** | 聂树斌 |
| --- | --- |
| **Individual Name (Romanized)** | Nie Shubin |
| **Gender** | Male |
| **Age at Conviction** | 20 |
| **Executed** | Yes |
| **Date of Execution** | Year: 1995 \| Month: 04 \| Day: 27 \| Precision: day |
| **Years Lost** | 0.6 |
| **Years Lost: Approximation Flag** | Yes (detention 23 September 1994 to execution 27 April 1995) |
| **Posthumous Case** | Yes (corrective action 2016, twenty-one years after execution) |

**C. Offense, Sentence, and Victim-Related Fields**

| **Offense Category** | Homicide; sexual offense |
| --- | --- |
| **Offense as Charged (Original Language)** | 故意杀人；强奸妇女 |
| **Offense as Charged (English Gloss)** | Intentional homicide; rape |
| **Sentence** | Death (immediate) |
| **Court of Original Trial** | Shijiazhuang City Intermediate People's Court (石家庄市中级人民法院); second instance: Hebei Higher People's Court (河北省高级人民法院) |
| **Evidence Relied Upon at Trial** | Confession (principal evidence); no independent forensic evidence linked the defendant to the crime |
| **Number of Victims** | 1 |
| **Victim Age Group** | Adult |
| **Victim–Defendant Relationship** | Unknown |
| **Alleged Victim Later Reappeared** | No |
| **Victim Identifying Details** | [Internal field — recorded; not displayed publicly] |
| **Actual Perpetrator Identified** | Yes |
| **Subsequent Victims Attributed to Actual Perpetrator** | Yes — the person who confessed was convicted of multiple rapes and murders committed in the same period |
| **Victim-Related Note** | One adult female victim, identified in public case records |

**D. Finality**

| **Finality Basis** | Ordinary appeals completed (second-instance judgment issued) |
| --- | --- |
| **Date of Finality** | Year: 1995 \| Month: — \| Day: — \| Precision: year |
| **Finality Note** | The Hebei Higher People's Court affirmed the death sentence at second instance; execution on 27 April 1995 confirms that the judgment had entered legal effect. |

**E. Official Post-Conviction Action**

| **Judicial Reversal (A)** | ☒ Marked |
| --- | --- |
| **Formal Acknowledgment of Error (B)** | ☒ Marked |
| **Innocence-Related Relief (C)** | ☐ Not marked |

**Official Actions List**

| **Field** | **Action 1** | **Action 2** |
| --- | --- | --- |
| Action Type | Retrial acquittal | State compensation decision |
| Category | A (Judicial reversal) | B (Formal acknowledgment of error) |
| Document Type | SPC Retrial Judgment (最高人民法院再审判决书) | State Compensation Decision (国家赔偿决定书) |
| Date of Action | Year: 2016 \| Month: 12 \| Day: 02 \| Precision: day | Year: 2017 \| Precision: year |
| Issuing Authority | Supreme People's Court (SPC) (最高人民法院) | Hebei Higher People's Court, Compensation Committee (河北省高级人民法院赔偿委员会) |
| Procedural Route | Retrial (再审) under the trial supervision procedure (审判监督程序) | State Compensation Law (国家赔偿法) |
| Basis Stated in the Decision | Insufficiency of the original evidence | Not stated |
| Summary | The SPC reversed the conviction in its entirety and declared Nie Shubin not guilty (宣告无罪), finding the evidence insufficient to establish guilt beyond reasonable doubt | RMB 2,684,600 awarded to Nie Shubin's parents; the decision is expressly premised on the wrongful conviction |
| **Compensation Awarded** | Yes |  |
| **Amount / Currency / Year** | 2,684,600 / CNY / 2017 |  |
| **Issuing Authority** | Hebei Higher People's Court (Compensation Committee) |  |
| **Decision Acknowledges Wrongful Conviction** | Yes (recorded as Category B, Action 2) |  |

**F. Case Summary**

**Background and Original Conviction.**

On 5 August 1994, the body of an adult woman was found in a cornfield in the western suburbs of Shijiazhuang, Hebei Province; she had been raped and murdered. Nie Shubin, then 20, was detained on 23 September 1994 and convicted of intentional homicide and rape by the Shijiazhuang City Intermediate People's Court; the Hebei Higher People's Court affirmed the death sentence at second instance. He was executed on 27 April 1995, approximately eight months after detention. The conviction rested principally on his confession; no independent forensic evidence linked him to the crime. Nie retracted the confession at trial, stating it had been obtained through coercion during six days of detention without access to counsel; the retraction was not accepted and no investigation into the alleged coercion was conducted.

**Emergence of Grounds for Correction.**

In 2005, Wang Shujin (王书金) was arrested for a series of rapes and murders and confessed, unprompted, to the 1994 offense, providing non-public details consistent with the crime scene. He maintained the confession and sought to be convicted of the offense in court. Prosecutors declined to charge him with it, citing Nie Shubin's existing conviction; the resulting deadlock lasted eleven years.

**Path to Official Action.**

Nie Shubin's mother, Zhang Huanzhi (张换枝), petitioned for reinvestigation for more than a decade, with growing public attention. On 28 April 2016 the SPC designated the case for retrial under the trial supervision procedure, and on 2 December 2016 it acquitted Nie Shubin on all charges, finding the evidence insufficient to exclude reasonable doubt. State compensation of RMB 2,684,600 was subsequently awarded to his parents. The case is cited in official and academic commentary as a landmark posthumous correction and a catalyst for reform of retrial procedure and pre-trial detention practice.

**G. Contributing Factors**

| **Factor / subcategory** | **Marked** | **Factual note (documentary basis)** |
| --- | --- | --- |
| 1. False or coerced confession | ☒ | Conviction rested principally on the confession; retracted at trial; the retrial judgment found the evidence, including the confession, insufficient to support guilt |
| 1a. Torture or physical coercion | ☒ | Physical abuse alleged in family and documented accounts; no interrogation recording existed by which the allegations could be verified (cross-coded to factor 4) |
| 1b. Prolonged detention or questioning without counsel | ☒ | Six days of detention without access to counsel before the confession |
| 1c. Deception, inducement, or threats | ☐ | — |
| 2. Eyewitness misidentification | ☐ | — |
| 3. Forensic or evidentiary error | ☐ | — |
| 4. Investigative or prosecutorial misconduct | ☒ | See subcategory below |
| 4a. Suppression of exculpatory evidence | ☐ | — |
| 4b. Failure to act on exculpatory developments | ☒ | Prosecutors declined to charge the confessing perpetrator with the offense, citing the existing conviction; documented in court records; prolonged the wrongful conviction for eleven years |
| 4c. Fabrication of evidence | ☐ | — |
| 5. Inadequate defense | ☒ | Confession not successfully contested at trial; limited pre-trial access to counsel and restricted disclosure under the procedure of the period |
| 6. Perjury or false accusation | ☐ | — |
| 7. Systemic or structural issue | ☒ | Mid-1990s procedural context: confession-dependent investigation, pre-trial detention practices, and institutional pressure for case resolution (He, 2016; Zhong & Dai, 2019) |
| 8. Other contributing factor | ☐ | — |

**H. Key Triggers of Official Action**

| **Trigger (controlled vocabulary)** | **Marked** | **Note** |
| --- | --- | --- |
| Real perpetrator identified | ☒ | Wang Shujin, 2005 |
| Confession by another person | ☒ | Wang Shujin's detailed, non-public-fact confession |
| Sustained petitioning or family advocacy | ☒ | Zhang Huanzhi, over more than a decade |
| Media or public attention | ☒ | Sustained national coverage preceding the 2016 retrial designation |
| All other permitted values | ☐ | — |

**I. Inclusion Verification (published in simplified form)**

| **Criterion** | **Status** | **Basis** |
| --- | --- | --- |
| Criminal offence | PASS | Intentional homicide and rape; criminal conviction within scope |
| Finality | PASS | Final under the two-instance system before the corrective action; execution confirms entry into legal effect |
| Official post-conviction action | PASS | SPC retrial acquittal, 2 December 2016 |
| Judicial Reversal (A) | MARKED | Retrial acquittal reversed the conviction in its entirety |
| Formal Acknowledgment of Error (B) | MARKED | State compensation decision expressly premised on wrongful conviction |
| Innocence-Related Relief (C) | NOT MARKED | No distinct relief instrument beyond the actions above |
| Documentation | PASS | Primary official sources listed in Section J |
| Inclusion determination | INCLUDED | Marked under Categories A and B |
| Codebook version at coding | v0.3 | — |

Coding memorandum: internal; not published.

**J. Supporting Documentation and Sources**

| **Source type** | **Title (original / English)** | **Authority** | **Date** | **Availability** |
| --- | --- | --- | --- | --- |
| Judgment | 最高人民法院再审判决书 / SPC Retrial Judgment | Supreme People's Court (SPC) | 2016-12-02 (day) | Published in the official judicial gazette (人民法院公报) |
| Compensation decision | 国家赔偿决定书 / State Compensation Decision | Hebei Higher People's Court (Compensation Committee) | 2017 (year) | Official record |
| Judgment | 原一、二审判决书 / Original first- and second-instance judgments | Shijiazhuang City Intermediate People's Court; Hebei Higher People's Court | 1995 (year) | Underlying conviction documents |
| Judgment | 王书金案相关审判文书 / Wang Shujin case documents | Hebei Higher People's Court | 2015 (year) | Documents the confession and the non-charging decision |
| Academic publication | He (2016), China Review; Zhong & Dai (2019), Journal of Contemporary China | — | 2016; 2019 | Supporting context |
